# The Importance of the Right Framework: Mitogen-Activated Protein Kinase Pathway and the Scaffolding Protein PTPIP51

**DOI:** 10.3390/ijms19103282

**Published:** 2018-10-22

**Authors:** Eric Dietel, Alexander Brobeil, Stefan Gattenlöhner, Monika Wimmer

**Affiliations:** 1 Institute of Anatomy and Cell Biology, Justus-Liebig-University, 35392 Giessen, Germany; monika.wimmer@anatomie.med.uni-giessen.de; 2 Institute of Pathology, Justus-Liebig-University, 35392 Giessen, Germany; alexander.brobeil@patho.med.uni-giessen.de (A.B.); stefan.gattenloehner@patho.med.uni-giessen.de (S.G.); 3 Institute of Anatomy, Johannes-Kepler-University Linz, 4040 Linz, Austria

**Keywords:** mitogen-activated protein kinase pathway (MAPK pathway), protein tyrosine phosphatase interacting protein 51 (PTPIP51), protein-protein interaction (PPI), cancer signaling

## Abstract

The protein tyrosine phosphatase interacting protein 51 (PTPIP51) regulates and interconnects signaling pathways, such as the mitogen-activated protein kinase (MAPK) pathway and an abundance of different others, e.g., Akt signaling, NF-κB signaling, and the communication between different cell organelles. PTPIP51 acts as a scaffold protein for signaling proteins, e.g., Raf-1, epidermal growth factor receptor (EGFR), human epidermal growth factor receptor 2 (Her2), as well as for other scaffold proteins, e.g., 14-3-3 proteins. These interactions are governed by the phosphorylation of serine and tyrosine residues of PTPIP51. The phosphorylation status is finely tuned by receptor tyrosine kinases (EGFR, Her2), non-receptor tyrosine kinases (c-Src) and the phosphatase protein tyrosine phosphatase 1B (PTP1B). This review addresses various diseases which display at least one alteration in these enzymes regulating PTPIP51-interactions. The objective of this review is to summarize the knowledge of the MAPK-related interactome of PTPIP51 for several tumor entities and metabolic disorders.

## 1. Background

The mitogen-activated protein kinase (MAPK) pathway is one of the best described signaling system in cancer. Almost one third of all human cancers have reported alterations in MAPK signaling, indicating the high relevance of the precise understanding of this pathway [1]. The basic role of the MAPK pathway is to transduce extracellular signals into the cell to regulate fundamental cellular functions including growth, cell migration, differentiation, and apoptosis [2]. To achieve a correct regulation of these diverging functions several distinct pathways are necessary [2]. The MAPK signaling consists of three different signaling systems, the extracellular signal-regulated kinase (ERK) pathway, the C-Jun N-terminal kinase/stress-activated protein kinase (JNK/SAPK) pathway and the p38 kinase pathway [3]. Each of these different signaling systems is strictly hierarchically structured and consists of a MAPK kinase kinase (MAPKKK), which is superior to a MAPK kinase (MAPKK), which controls a MAPK [3]. Of these different systems, the ERK pathway is the best studied MAPK pathway. The ERK signaling can be activated by numerous extracellular stimuli, e.g., growth factors or mitogens. One example for a classical activation path is represented by the activation of the epidermal growth factor receptor (EGFR). Its hetero- or homodimerization induced by binding of epidermal growth factors leads to an autophosphorylation of the receptor [4]. Consequently, a signaling cascade consisting of growth factor receptor-bound protein 2 (GRB2), son of sevenless (SOS), and the small GTPase Ras is activated. GTP-bound Ras recruits Raf kinases to the cell membrane for activation [4]. The Raf kinases represent the MAPKKK in the ERK pathway. Subsequently, Raf kinases activate MEK1/2 (MAPKK) and ERK1/2 (MAPK) [2]. The targets of ERK1/2 are diverse and include p90^RSK^, mitogen-activated protein kinase interacting protein kinases 1 and 2 (MNK1/2), Ets, Ets domain-containing protein (Elk1), Myc, signal transducer and activator of transcription 1/3 (STAT1/3) and estrogen receptor (ER), to name some of them [2]. These many targets are necessary for the precise regulation of the various aforementioned cellular functions, e.g., differentiation, growth, apoptosis, and migration.

Since, the MAPK pathway controls these essential functions a precise regulation and titration of the signaling activity is needed. A perfect example for such a strict regulation is the upstream positioned Raf kinases. The Raf kinase family consists of three different Raf proteins, Raf-1, B-Raf and A-Raf. Although their structures are almost similar, their activation modes are extremely different. After recruitment of Raf kinases to GTP-bound Ras, a complex series of phosphorylations is induced for activation. These phosphorylations are needed for the activation of the kinase domain and reduction of the autoinhibition. The activation of Raf-1 and A-Raf requires phosphorylation of the N-region, dephosphorylation of the S259 inhibitory site, and phosphorylation of the activation loop. B-Raf is already in a preactivated state and can be fully activated by Ras alone, whereas the activation of Raf-1 and A-Raf requires other factors [1]. Due to this, only small aberrations in the structure of B-Raf, such as the V600E exchange, are needed to induce a constitutive activation. Alterations such as these are found in almost two thirds of malignant melanoma and in glioblastoma, where of more than 40 different mutations in the B-Raf gene 90% are at residue 600 in exon 15 [5].

Another regulating mode of the Raf kinases is the binding to scaffolding proteins such as the 14-3-3 protein family [6]. Such adaptor and scaffolding proteins facilitate the correct subcellular localization, provide a proximity of different signaling partners and support the formation of multiprotein complexes [3]. Moreover, scaffolding proteins can shield activated signaling molecules from deactivating phosphatases to allow an adequate signaling strength [3]. Additionally, scaffolds provide crosstalks between different signaling pathways.

**Figure 1:**
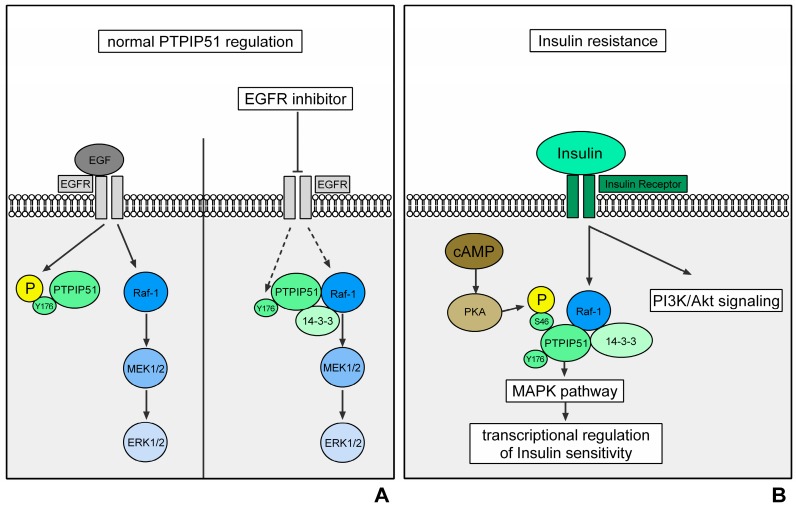
Regulation of PTPIP51 interactions in normal cells (represented by the HaCat cell line). Activation of the EGFR via the binding of EGF induces an activation of Raf-1 via several signaling molecules. Raf-1 depicts the MAPKKK of the ERK signaling. Its activation triggers a signaling cascade via MEK1/2 and ERK1/2, which ultimately initiates transcription. The EGFR also phosphorylates the Tyr176 residue of PTPIP51 and thereby inhibits its interaction with Raf-1. This mechanism prevents an overshooting activation of the MAPK pathway. The right side of the figure represents the interactions when EGFR is inhibited. The inhibition of EGFR leads to an omission of Tyr176 phosphorylation of PTPIP51 via the EGFR. The dephosphorylation of PTPIP51 at Tyr176 induces the formation of the Raf-1/14-3-3β/PTPIP51 complex and thus a stimulation of the MAPK pathway. This mechanism partially compensates for the EGFR inhibition (black arrows indicate a phosphorylation/activation; dotted black arrows indicate a reduced phosphorylation/activation) (**A**); regulation of PTPIP51 interactions in insulin resistance. Activation of the insulin receptor induces the activation of the PI3K-Akt-mTOR signaling and the MAPK pathway, especially the ERK signaling. Here, PTPIP51 stimulates the signaling on Raf-1 level and potentially modulates the insulin sensitivity on transcriptional level. Protein kinase A (PKA) phosphorylates the Ser46 residue of PTPIP51 and thereby stimulates the binding of PTPIP51 and Raf-1 via 14-3-3β (black arrows indicate a phosphorylation/activation) (**B**).

Of note, the protein tyrosine phosphatase interacting protein 51 (PTPIP51) represents another scaffold protein, which regulates MAPK activation on Raf-1 level [7]. PTPIP51 exerts its regulating effect on the MAPK pathway on Raf-1 level via the scaffold protein 14-3-3β [7]. The recruitment of PTPIP51 into the MAPK signaling leads to an activation of the MAPK pathway. A well-titrated signal is a prerequisite for an optimal cellular function. Therefore, the formation of the PTPIP51/14-3-3β/Raf-1 complex is tightly regulated by kinases and phosphatases [8,9]. One of the crucial spots for this regulation is the tyrosine 176 residue of PTPIP51 [9,10,11]. Its phosphorylation results in a break-up of the PTPIP51/14-3-3β/Raf-1 complex and hence the stimulation of the MAPK signaling is omitted [8,9,11,12]. The phosphorylation of the tyrosine 176 residue is under the control of the EGFR and other kinases, such as the cellular sarcoma kinase (c-Src) [8,10,11,12]. Dephosphorylation is mainly performed by PTP1B [9,11,12]. Another important phosphorylation site of PTPIP51 is the serine 212 residue. Computational models of the PTPIP51 molecule show a cleft in its tertiary structure, which is surrounded by the aforementioned tyrosine 176 residue and serine 212 residue, respectively [9]. Up to now, we assume, that the cleft represents a binding site for the Raf kinases [9]. Contrary to the interaction inhibiting tyrosine 176 residue, phosphorylation of the serine 212 residue leads to an augmentation of the interaction with Raf-1 via 14-3-3β [7,8,9,12]. Besides the cleft, PTPIP51 protein structure contains tetratricopeptide domains, which are known to serve as binding sites for protein-protein interactions [9]. Additionally, in the structure of PTPIP51 two conserved regions are found. These sites facilitate the interaction with the scaffolding protein 14-3-3β [7,9]. In summary, PTPIP51 possesses the perfect scaffolding protein equipment, encompassing several binding sites for protein-protein interactions and the capability of modulating these bindings via phosphorylation and dephosphorylation of tyrosine and serine residues (Figure 1A).

Besides the direct regulation of the MAPK pathway, PTPIP51 is involved in a broad range of cellular functions and signaling systems. The panel of interaction partners ranges from NF-κB signaling proteins (RelA, I-κB) over mitochondrial associated ER membrane-related proteins (VAPB, ORP5/ORP8), autophagy-related signaling, and mitosis associated proteins (CGI-99, Nuf2) [13,14,15,16,17,18,19,20]. These interactions of PTPIP51 are already reviewed and analyzed by studies of our group and other scientists. Therefore, the focus of this review is to highlight the regulation of PTPIP51 and its functional consequences affecting the MAPK signaling in diseases associated with an aberrant MAPK signaling.

## 2. Regulation of PTPIP51 in Metabolic Signaling

### 2.1. Insulin Resistance and Obesity

Obesity and insulin resistance and their secondary diseases have reached epidemic proportions. More than one third of the US population has a diagnosed obesity and 10% of the adults are affected of diabetes [21]. The annual medical costs to manage these diseases are estimated to exceed $245 billion showing not only the medical but also the economical relevance of the precise understanding of the underlying mechanisms of obesity and insulin resistance to develop an effective therapy [21].

The most important signaling pathways for the regulation of lipid storages in adipocytes are the insulin receptor and the PKA signaling, which act as antagonists [22]. Whereas insulin receptor activity leads to lipogenesis, the PKA activation induces lipolysis [22,23,24]. Insulin receptor activation is mediated by binding of insulin and subsequently the autophosphorylation of the receptor molecules [24]. This results in the binding of insulin receptor substrates, which ultimately induce the activation of the two most important downstream signaling pathways, the PI3K-Akt-mTOR pathway and the ERK pathway [24]. While PI3K-Akt-mTOR signaling controls the lipid synthesis, glycogen synthesis, expression of metabolism-related proteins and glucose transport, the ERK pathway is needed for proliferation signaling [24]. The activation of ERK signaling also provides a mechanism for the adjustment of insulin sensitivity [25,26]. Zhang and coworkers showed, that the MAPK pathway controls the expression of the insulin-like receptor via the ETS-1 transcription factor pointed in Drosophila, thus ensuring the correct insulin sensitivity for the maintenance of adequate glucose levels [25].

The activity level of the insulin receptor is not only determined by the presence of insulin, but also by the phosphorylation status of the receptor. The phosphatase PTP1B represents a crucial downregulator of insulin receptor activity [24,27]. The impact of PTP1B on insulin-related signaling is mirrored by the results of PTP1B knockout models [28,29]. PTP1B knockout mice do not develop obesity or insulin resistance if fed a high-fat diet [28,29]. Thus, the inhibition of PTP1B via small molecule inhibitors seems a promising therapeutic strategy, but a safe and selective PTP1B inhibitor has yet to be identified [27].

#### PTPIP51 in Insulin Resistance and Obesity

The expression of PTPIP51 in adipocytes is tightly regulated by the diet and the submission to training in mice [22]. Bobrich and coworkers showed, that the expression of PTPIP51 protein correlates with the grade of insulin sensitivity, whereas the adipose tissue of normal mice contains the highest amount of PTPIP51 protein and that of high-fat diet-fed mice the lowest amount [22]. Mice fed a high-fat diet and subjected to training expressed levels of PTPIP51 protein lying in between the two aforementioned groups. Interestingly, also the interaction of PTP1B and PTPIP51 is correlated with the training and to the diet [22]. High interaction levels of PTPIP51 and PTP1B as seen in normal and trained mice ensure the stimulating effect of PTPIP51 on MAPK pathway via dephosphorylation of the PTPIP51 Tyr176 residue and the formation of the Raf-1/14-3-3β/PTPIP51 complex [22]. Subsequently, a similar insulin sensitizing effect via transcriptional control of the insulin receptor as cited above for Drosophila could be possible [25]. This hypothesis is supported by the upregulated interaction of 14-3-3β and PTPIP51 in high-fat diet, trained mice [22]. Here, the stimulation of the MAPK pathway by PTPIP51 could be a bypassing mechanism for the high-fat diet induced insulin receptor resistance.

PTPIP51 also interacts with the insulin receptor signaling antagonist, the PKA [8,9]. PKA is a serine/threonine kinase, which is activated by high cAMP levels in consequence of extracellular signals [30]. PKA phosphorylates PTPIP51 at serine 46 and serine 212 in vitro [10]. Additionally, using the Group-based Prediction System 3.0 (http://gps.biocuckoo.org/), PKA was identified to phosphorylate the serine residue 149 of PTPIP51 [9]. Interestingly, the serine residues 46 and 149 are in direct vicinity of the two conserved regions of PTPIP51 [9]. The serine residue 212 is located close to the cleft of PTPIP51, which presumably depicts the binding pocket for Raf-1 [9]. Thus, PKA can modulate the binding of PTPIP51 to 14-3-3 proteins and Raf-1 [8,9,11]. Here, PTPIP51 represent a possible mediator between the katabolic PKA signaling and the anabolic insulin signaling.

The central position in metabolic signaling mirrors the important role of PTPIP51 in the genesis of insulin resistance and obesity. Up to now, PTPIP51 seems to maintain a level of insulin sensitivity via stimulation of MAPK signaling. As mentioned above this stimulating effect is active if PTPIP51 is dephosphorylated at the Tyr176 residue. This conflicts with the observed outstanding findings of PTP1B knockout mice, since PTP1B is essential for the dephosphorylation of PTPIP51 [8,9,10,11,12]. The functional and interactional consequences of PTP1B knockout or inhibition must be subject to further studies, as the aforementioned medical and economic implications strongly highlight the importance of the precise understanding of PTPIP51 in these signaling systems (Figure 1B).

## 3. Regulation of PTPIP51 in Cancer

### 3.1. Breast Cancer

Breast cancer is the most common neoplasm in women, accounting for about 25% of all diagnosed tumors. Although early diagnosis and enhanced therapies of breast carcinomas greatly improved the overall survival time, breast cancer is still the third most cause of cancer deaths in the US [31,32].

About one third of the breast tumors exhibit an overexpression of the HER2/ErbB2 receptor, leading to a more aggressive and invasive growth of the cancer cells and thus an impaired overall survival time [33]. The amplification of the Her2 receptor induces an over-activation of mainly two different signaling pathways, the MAPK pathway and the Akt signaling [34]. The activation of these signaling pathways is mediated by the enhanced formation of homodimers and heterodimers of the Her2 receptor and other members of the Her family, namely EGFR, Her2, and Her4 [34]. Subsequently, the signaling is channeled via the aforementioned signaling molecules into the ERK signaling, resulting in an enhanced growth and proliferation of the cancer cells [34,35].

The knowledge about Her2 receptor amplification and its functional consequences in breast tumors led to the development of several Her2 targeted therapies encompassing small molecule tyrosine kinase inhibitors and monoclonal antibodies [33,36]. Introduction of these substances prompted a great progress in the clinical management of Her2 amplified breast cancer [33,36]. For example, targeting the HER2 receptor with the monoclonal antibody trastuzumab improved the disease-free survival rates at 5 years from 75% to 81–84% in HER2-positive early stage breast cancer [37].

Despite the good clinical results, the management of anti Her2 therapy resistances are still challenging. Several signaling pathways have been identified to mediate these resistances, e.g., the PI3K-Akt-mTOR signaling, c-MET signaling pathway or low immune response [38,39,40].

The regulation of the non-receptor tyrosine kinase c-Src depicts another crucial resistance mechanism [41,42]. c-Src is involved in many cellular processes regulating cell proliferation and cell survival [43]. These functions are exerted via the interaction of c-Src with several receptor tyrosine kinases and other signaling hubs, e.g., the MAPK pathway and PI3K-Akt signaling [43]. Activation of c-Src is observed in tumors of the colon, liver, lung, and pancreas [41]. In breast tumors high levels of activated c-Src correlate with poor prognosis, lower overall survival time and trastuzumab resistance [40,41]. In fact, the activation of c-Src alone is capable of conferring trastuzumab resistance [44]. Thus, the combination of anti-Her2 and anti-c-Src therapies seems a promising concept for the treatment of resistant tumors. Actually, recent studies verified this approach. Combination of Saracatinib, a small molecule inhibitor of c-Src and Lapatinib, a small molecule inhibitor of Her2 resulted in prolonged survival in a xenograft mouse model [45]. Furthermore, a direct interaction of c-Src and Her family members is pivotal in Her2 amplified breast cancer cells for the exertion of mitogenesis upon EGF stimulation and the correct transduction of growth promoting effects of heregulin [46]. The physical interaction of Her2 and c-Src seems to be crucial for the transduction of survival and growth signals of Her2 heterodimers [46]. Furthermore, Her2/c-Src interaction promotes the anchorage-independent growth of Her2 amplified breast cancer cells [46].

For the precise titration of phosphorylation levels of receptor tyrosine kinases (RTKs), signaling kinases and scaffolding proteins not only kinases, such as c-Src, are needed but also phosphatases. One of the best described phosphatases is the protein tyrosine phosphatase 1B (PTP1B) [47]. PTP1B is involved in the modulation of several RTK, e.g., EGFR and Her2 [28]. The relevance of PTP1B in breast cancer is mirrored in its interaction with the Her2 receptor. Usually, PTP1B is involved in the dephosphorylation and thereby deactivation of RTKs. In contrast, the Her2 receptor is activated by PTP1B via an up to now unknown mode of action [28]. The combination of Her2 overexpression and PTP1B knockdown in a mouse model resulted in a delayed tumor development of about 85 days compared to mice with normal PTP1B expression [48]. Furthermore, inhibition of PTP1B in breast gland cells leads to a reduced proliferation, it also affects the epithelial-mesenchymal-transition, which is a hallmark in the formation of metastasis [49]. All these findings stress the importance of PTP1B in the development and the progression of breast tumors, especially in Her2 amplified breast cancer cells. A precise understanding of the PTP1B affected signaling pathways is of the utmost interest to provide a platform for the development of novel therapeutic strategies.

#### PTPIP51 in Breast Carcinoma

The scaffolding protein PTPIP51 represents a crucial crossing point of all the aforementioned tumor promoting and resistance inducing mechanisms. PTPIP51 is expressed in normal breast glands as well as in breast cancer cells, whereby the expression of PTPIP51 protein is diminished in the cancer cells (ongoing studies of our group). Further analysis of the crucial Tyr 176 phosphorylation site of PTPIP51 showed a strong upregulation of the phosphorylation in breast cancer cells. As shown by Brobeil et al. the Tyr 176 residue phosphorylation regulates the binding of PTPIP51 to Raf-1 via 14-3-3β and thereby exerts its MAPK stimulating effect [8]. The downregulation of PTPIP51 in combination with the high phosphorylation of the Tyr176 residue depicts a potential inhibition of the MAPK stimulating effect of PTPIP51 in breast cancer cells. Thus, the regulation of PTPIP51 seems to counteract the activation of the tumor promoting MAPK signaling.

In Her2 amplified breast cancer cells, the phosphorylation of PTPIP51 at Tyr176 is to a great extend performed by the EGFR. Inhibition of the EGFR in Her2 amplified breast cancer cells induces a reduction of PTPIP51 phosphorylation at the Tyr176 residue accompanied by a formation of the Raf-1/14-3-3β/PTPIP51 interactome, thus proofing a normal regulation of MAPK-related interactions of PTPIP51 [50].

Interestingly, PTPIP51 also interacts with the Her2 receptor, but it is not clear if the Her2 receptor phosphorylates PTPIP51 or if PTPIP51 forms a scaffold for the interaction of the Her2 receptor with other signaling molecules [50]. Of note, selective inhibition of the Her2 receptor with the TKI Mubritinib induces a formation of a potential ternary interactome consisting of the Her2 receptor, PTPIP51 and c-Src [50]. As mentioned above, c-Src plays a crucial role in Her2 targeted therapy resistance and the transduction of growth and survival signals [41,42,44,46]. The formation of the ternary complex Her2/c-Src/PTPIP51 stresses a pivotal role of PTPIP51 in the mediation of these resistance mechanisms [50].

In addition, own studies showed the relevance of the PTPIP51/c-Src interaction in correlation with the sensitivity of Her2 positive breast cancer cells to EGFR/Her2 targeted TKIs. Application of several EGFR/Her2 targeted TKIs to the Her2 amplified breast cancer cell line SK-BR3 led to a highly significant augmentation of PTPIP51/c-Src interaction, whereas the same treatment of BT474 cells, also a Her2 amplified breast cancer cell line, did not alter or even reduce the PTPIP51/c-Src interaction. Interestingly, the enhanced interaction of PTPIP51 and c-Src was accompanied by a less sensitivity to TKI treatment, representing another potential resistance mechanism [50].

Of note, as aforementioned the interaction of PTPIP51 and c-Src is severely altered in Her2 amplified breast cancer cells when treated with EGFR/Her2 targeted TKIs, but the interaction of PTPIP51 and its crucial phosphatase PTP1B remains nearly unaffected [50]. Solely long-term application of Gefitinib (EGFR TKI) and Lapatinib (EGFR/Her2 TKI) induced an upregulation of PTPIP51/PTP1B interactions [50], thus, portraying a potential adaption mechanism of the cancer cell to the applied TKI treatment and not a direct effect of the TKI. Interestingly, this adaption seems contradictory since the inhibition of EGFR results in a reduced Tyr176 phosphorylation and a compensation would be a downregulation of PTPIP51/PTP1B interaction. Moreover, the upregulation of PTPIP51/PTP1B interaction upon EGFR inhibition is accompanied by an increase in the sensitivity of SK-BR3 cells (Her2 amplified breast cancer cell line) to the treatment, which is not seen after short term application. Interestingly, these regulations of PTPIP51/PTP1B interaction do not occur under selective Her2 inhibition [50]. Up to now it is not clear but seems probable whether there is a link between the aforementioned differing effects of PTP1B on EGFR and Her2 activity and the diverging effects of EGFR inhibition and selective Her2 inhibition on the PTPIP51/PTP1B interaction. Interestingly, the application of the EGFR/Her2 TKI Neratinib did not reduce the Tyr176 phosphorylation of PTPIP51 to the same extend as Gefitinib and Lapatinib. This regulation is accompanied by a reduced interaction of PTPIP51 and PTP1B. Thus, the reduced phosphorylation by EGFR is counter regulated via a reduced dephosphorylation of PTPIP51 through PTP1B under Neratinib treatment.

Additionally, the importance of the PTPIP51/PTP1B interaction is underlined by the fact, that this interaction is directly correlated with the grading of breast carcinomas. Ongoing studies of our group examined the interaction of PTPIP51 and PTP1B in breast carcinomas of no special type and showed a significant enhancement of the interaction in grade 3 carcinomas compared to grade 1 and 2 carcinomas. Moreover, Her2 amplified carcinomas also displayed a significantly upregulated PTPIP51/PTP1B interaction compared to Her2 negative breast cancer samples. This may be supported by the upregulation of PTP1B in Her2 amplified breast cancer.

**Figure 2:**
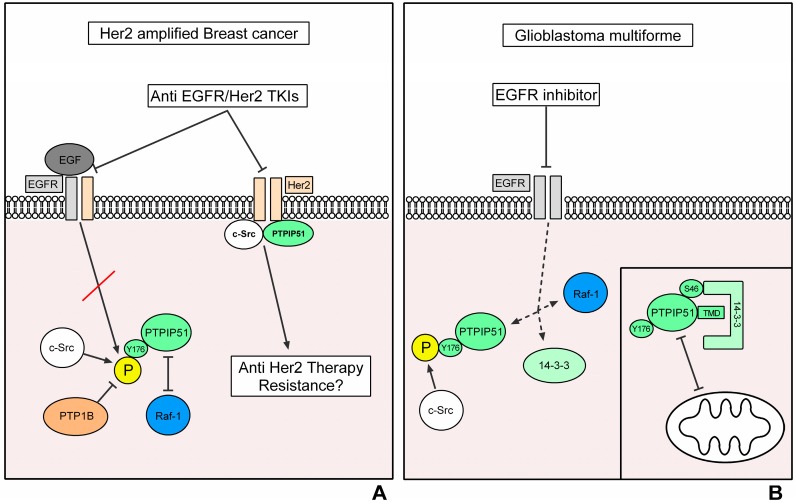
Regulation of PTPIP51 interactions in breast cancer. The inhibition of EGFR in Her2 amplified breast cancer cells induces the same effects as observed in the HaCat cell line upon EGFR inhibition regarding the formation of the Raf-1/14-3-3β/PTPIP51 complex. The sensitivity of Her2 amplified breast cancer cells towards EGFR-targeted TKIs correlates with the regulation of the interaction of PTPIP51 with c-Src. The selective inhibition of Her2 induces a formation of a PTPIP51/Her2/c-Src complex, which depicts a potential resistance mechanism against anti-Her2 therapies (black arrows indicate a phosphorylation/activation, arrows with vertical bar as arrow head indicate an inhibition of interaction/activation) (**A**); regulation of PTPIP51 interactions in Glioblastoma multiforme. The left side of the figures depicts the regulation of PTPIP51 interactome under EGFR inhibition. Contrary to the expectations, the inhibition of the EGFR induces a disruption of the Raf-1/14-3-3β/PTPIP51 complex. The right side of the figure shows that the upregulated 14-3-3 protein levels in gliomas of high malignancy potentially inhibit the translocation of PTPIP51 to the mitochondrion and thus its apoptosis-inducing effects (black arrows indicate a phosphorylation/activation, arrows with vertical bar as arrow head indicate an inhibition of interaction/activation, dotted black arrows indicate a dissolution of the Raf-1/14-3-3β/PTPIP51 complex via EGFR inhibition) (**B**).

To sum up, PTPIP51 is needed for the normal function of healthy mammary glands and its expression is altered in the development of breast tumors. Furthermore, the interaction of PTPIP51 and PTP1B correlates with the grading and the Her2 amplification, indicating an alteration of PTPIP51 phosphorylation during the progression of breast carcinoma. We were able to unveil a potential role of PTPIP51 in tumor promoting signaling and therapy resistance against EGFR/Her2 targeted TKIs mediated through the non-receptor kinase c-Src and the phosphatase PTP1B [50]. In consequence, PTPIP51 plays a pivotal role in the oncogenesis of breast carcinoma and it is of the utmost interest to unveil the regulations of PTPIP51 in respect of therapy resistance and growth signaling (Figure 2A).

### 3.2. Glioblastoma Multiforme

One of the most malignant tumors with an overall survival time of about one year, despite significant advances in the therapeutic options, is the glioblastoma multiforme (GBM) [51,52,53]. The GBM represents the most common primary brain tumor in adults, accounting for almost 80% of all primary brain tumors [52,53]. The clinical presentation depends on the location and the size of the GBM and ranges from headache over neurological deficits to seizures, which are present in about 25% of the patients at the time of diagnosis [52]. The multimodal treatment of GBM includes radical surgical resection, radiotherapy, and chemotherapy. Despite the extensive clinical management, the prognosis of GBM is poor and a curation is not possible [52,54]. Due to this the Cancer Genome Atlas project and the genomic profiling of more than 600 genes of 200 human tumor samples were performed to get an insight in the most frequent signaling aberrations in GBM. The three most commonly activated signaling pathways were the p53 signaling, the retinoblastoma pathway and the receptor tyrosine kinase-Ras-PI3K pathway [55]. One special receptor tyrosine kinase in GBM is the EGFR, respectively, its constitutively activated mutant, the EGFRvIII [53,56,57]. This mutation occurs in about 30% of all GBM [58]. The constitutive activation of the EGFRvIII leads to an over-activation of the MAPK pathway and thereby an exaggerated growth and survival signaling [58]. Targeting the aberrant EGFR and thereby blocking the aggressive growth of GBM seems a reasonable therapy strategy, but several clinical trials demonstrated only discrete improvements of a small percentage of GBM patients, when treated with EGFR inhibitors [57]. The most recent strategy is a vaccination against the EGFRvIII, which seemed a promising approach. However, again the theory did not approve in the clinical setting [52].

Consequently, many GBMs must have an intrinsic or a rapidly acquired resistance against EGFR inhibitors or the signaling is bypassed via alternative growth and survival promoting pathways [59]. Therefore, it is of the utmost interest to unveil the downstream pathways of the EGFR in GBM.

The most commonly activated signaling pathway downstream of the EGFRvIII is the ERK signaling [60]. The activated EGFRvIII induces an activation of Raf-1 by signaling molecules mentioned in the background section. Subsequently, the activation of Raf-1 facilitates the activation p90^RSK^, MNK1/2, Ets, Elk1, Myc, STAT1/3, which mediate the proliferation and growth inducing effects of the EGFRvIII activation [2]. A crucial regulator of Raf-1 is the scaffolding protein 14-3-3β [6,7]. 14-3-3 proteins belong to a highly conserved protein family and are expressed in all human cells. Due to the lack of a kinase domain, 14-3-3 proteins exert their function via binding of serine phosphorylated proteins [61]. Thereby, 14-3-3β ensures proximity of signaling substrates and provides a corresponding reaction matrix [61]. Furthermore, for the activation of some signaling molecules 14-3-3 proteins are of utmost need as seen for the activation of Raf-1 [6]. Thorson and coworkers reported, that an activation of Raf-1 in the absence of 14-3-3β is not possible. The addition of 14-3-3β restored the activation of Raf-1 [6].

In the setting of GBM 14-3-3 proteins also seem to play a crucial role. Yang and coworkers showed a direct correlation between the amount of expressed 14-3-3β and 14-3-3η and the grade of malignancy of glioma [62]. The importance of 14-3-3 proteins in GBM is further mirrored by their mediation of radio- and chemotherapy resistance. Park and coworkers found, that the depletion of 14-3-3η enhances the radiosensitivity of GBM cells [63]. Similar results were found in earlier studies concerning the sensitivity of GBM to chemotherapeutics, such as microtubule agents [63].

The examination of the aforementioned cellular signaling and many more, e.g., platelet-derived growth factor receptor (PDGFR) signaling, neurofibromatosis type 1 (NF1) and the tumor microenvironment, lead to crucial insights in the aberrant tumor signaling, but up to now a linkage between these structures is still missing.

#### PTPIP51 in Glioblastoma

PTPIP51 depicts a possible functional linkage between the constitutively activated EGFRvIII and the scaffolding protein 14-3-3β. PTPIP51 is expressed in glioma of low malignancy as well as in GBM [64]. The expression of PTPIP51 protein and mRNA directly correlates with the grade of malignancy and thus mirrors the expression of 14-3-3 proteins in primary brain tumors [64]. The upregulation of 14-3-3β and 14-3-3η is associated with reduced apoptosis [63]. Of note, PTPIP51 includes an N-terminal transmembrane domain (TMD), when expressed in its full form. The TMD is responsible for the translocation of PTPIP51 towards the mitochondrion. Lv and coworkers showed, that the overexpression of PTPIP51 leads to an accumulation at the mitochondrion and subsequently to the induction of apoptosis [65]. Besides the apoptosis-related functions, mitochondrion located PTPIP51 is involved in the correct formation of mitochondrion-related endoplasmic reticulum membranes (MAM) and the calcium signaling between mitochondrion and endoplasmic reticulum, but these functions are not in the scope of this review [13,14,15,16].

Interestingly, the PTPIP51 protein structure includes two conserved regions, which facilitate the binding to 14-3-3 proteins [7,9]. Moreover, these sites are accompanied by serine and tyrosine residues, whose phosphorylation control the binding of PTPIP51 and 14-3-3β [9]. The conserved region 1, which spans from aas 43 to aas 48 is near the TMD [9]. Binding of 14-3-3β at the conserved region 1 leads to a capping of the TMD and thus the translocation of PTPIP51 towards the mitochondrion is abrogated [9,12]. In consequence, the hindrance of the translocation of PTPIP51 to the mitochondrion via binding of the upregulated 14-3-3 proteins in gliomas of high malignancy may depict a potential apoptosis resistance mechanism.

As aforementioned, the inhibition of the constitutively activated EGFRvIII seemed a promising therapeutic strategy, but up to now the clinical results lack a sufficient improvement of the patients’ outcome [52]. Interestingly, the inhibition of EGFR with TKIs in glioblastoma cells leads to completely unexpected regulations in the MAPK-related interactome of PTPIP51. Usually, the inhibition of the EGFR induces a reduction of Tyr176 phosphorylation of PTPIP51 and consequently to a formation of the Raf-1/14-3-3β/PTPIP51 complex, which at least partially compensates for the reduced MAPK pathway activation [8,9,10,11,12]. In glioblastoma cells the regulations upon EGFR inhibition differ. Duolink proximity ligation assays revealed a decrease in the interaction of 14-3-3β/PTPIP51 and Raf-1/PTPIP51, indicating an omission of the PTPIP51 mediated MAPK pathway stimulating effect. Accordingly, the phosphorylation of Tyr176 of PTPIP51 was only slightly altered [66]. A possible explanation of these regulations is an overshooting compensation for the loss of the EGFR activity by c-Src activation [66]. c-Src is frequently overexpressed and over-activated in gliomas of high malignancy [67]. Likewise, mechanisms were seen in Her2 amplified breast cancer cells. Here, c-Src/PTPIP51 interaction directly correlated with the resistance towards EGFR-targeted therapies [50]. Thus, in tumors with an altered RTK signaling the interaction of PTPIP51 and c-Src may constitute a tumor-interspecies mechanism of anti EGFR therapy resistance (Figure 2B).

### 3.3. Melanoma

Melanomas belong to a cancer entity arising from melanocytes originally derived from neural crest cells found in skin and uvea. Among the three main types of skin cancer, beside melanoma there are basal cell carcinoma and squamous cell carcinoma. The melanomas account for about 2% of skin cancers, but for 75% to 90% of deaths [68]. Melanomas display the highest mutation frequency of all cancers [69]. About 2.3% of people will develop a melanoma during life time. In 2015 an estimated 1.2 million Americans were living with a melanoma (NIH National Cancer Institute, Surveillance, Epidemiology, and End Results https://seer.cancer.gov/statfacts/html/melan.html). From all cancers melanomas have the highest probability to metastasize to the brain [70]. In 50% of patients dying from melanoma the reasons are brain metastasis [71].

For the pathogenesis of melanomas, the activation of the MAPK pathway is essential [72]. This is reflected by genetic alterations in melanomas affecting molecules linked to the MAPK signaling. Possible candidates are the BRAF kinase, the small GTPase NRAS and the c-KIT receptor. The BRAF mutations are present in about 40–50%, mutations of NRAS in 20% and of c-KIT in 1–3% of the melanomas [69,73]. Metastatic melanomas are subdivided by their mutation profile into four subtypes BRAF driven (about 52%), NRAS driven (28%), *Neurofibromin 1 (NF1)* mutated (14%) and in “triple wild type” [74]. The knowledge of these specific mutations within the MAPK pathway is essential for therapeutic options.

CRAF/Raf-1 is expressed in all cells, but BRAF is associated with cells of neuronal origin such as melanocytes. CRAF mutations are rare in contrast to BRAF mutations [75]. BRAF mutations seen in melanomas are MAPK pathway activating mutations. Commonly 80 to 90% of the BRAF mutations are a single amino acid substitution in the BRAF protein where valine is substituted by glutamic acid at position 600 (BRAF V600E). In 15% of BRAF mutated melanomas valine is substituted by lysine (BRAF V600K) and in 3% of the cases by arginine (V600R) or by aspartic acid (V600D). Yet, any of these mutations leads to a constitutively activated BRAF kinase as the mutation is in the activation loop of the kinase [76]. Due to this modification within the activation segment of BRAF no extracellular signal is needed for upregulated MAPK signaling by increased MEK phosphorylation which in turn phosphorylates ERK stimulating downstream signaling and resulting in proliferation [77]. The knowledge of these mutations prompted new therapy options by targeting the overactive BRAF kinase through specific inhibitors such as vemurafinib and dabrafenib, which bind to the active site of BRAF kinase, encorafenib an ATP-competitive Raf kinase inhibitor and sorafenib, a multi-kinase inhibitor [76].

For example, vemurafinib treated patients showed an overall response rate of 52.2% and the median progression-free survival was 8.3 months; the median overall survival was 13.5 months according to a study of Si and coworkers [78]. This form of therapy is also challenged by acquired resistance, despite initial good results.

Mechanisms promoting resistances can be manifold: a reactivation of MAPK signaling (amplification or activation of target kinases); bypass via different signaling pathways, e.g., PI3K-Akt-mTOR signaling; or by the surrounding stromal cells, which secrete HGF in BRAFi-resistant melanomas, activating MAPK and PI3K-Akt-mTOR.

Next to the more common BRAF oncogene, mutations in the NRAS gene result in more aggressive melanomas [76]. BRAF mutations can effectively be targeted by specific small molecule inhibitors, whereas NRAS-mutated subtypes are more susceptible to immunotherapy [79]. NRAS mutations are seen in all types of melanomas but seem to be slightly more numerous in melanomas of sun-damaged skin [80]. The NRAS gene belongs to the Ras oncogene family coding for the NRAS protein a GTPase, which is activated by bound GTP. When activated, NRAS binds to Raf and changes its conformation, thus activating the Raf kinase stimulating the MAPK pathway.

NRAS mutations lead to a substitution of the amino acids at positions G12 or G13 making NRAS insensitive to the inactivation by the Ras GTPase-activating proteins, or more common at position Q61, where Q61R is predominant in melanoma cells [81]. Ras mutations at position Q61 are associated with impaired GTPase activity, thus NRAS is constitutively activated due to its GTP-associated conformation [82].

As there exist no specific NRAS/Ras inhibitors, different strategies for treatment of NRAS-mutated melanomas were administered for therapy. The use of farnesyl transferase inhibitors to prevent posttranslational modification of Ras and its insertion into the plasma membrane failed in clinical studies [83]. Third generation MEK1/2 inhibitors such as binimetinib (MEK162)-a potent allosteric inhibitor, improved the response rate and progression-free survival of patients, but failed to prolong overall survival of the patients [84].

The first immunotherapies successfully approved interleukin 2 and anti-CTLA4 antibody both stimulating the immune response as treatment options [84]. As seen in clinical trials 25 to 50% of patients respond to the inhibition of the immune checkpoint PD1 [84]. In 2016 Johnson and coworkers already stated, that NRAS mutations displayed a superior clinical outcome in immune therapy compared to BRAF mutations [85]. This better response of NRAS-mutated melanoma to immune therapy is due to a higher level of immunosuppression in the tumor microenvironment compared to BRAF-mutant melanoma [86]. Immune therapies of NRAS-mutated melanomas are now the first-line treatment for NRAS and WT melanoma [84]. Immune checkpoint blockade inhibitors significantly improved overall survival rates [87]. Yet, eventually any of these treatments leads to a resistant behavior of the tumor cells. Echevarrı′a-Vargas et al. addressed this problem by a new therapeutic strategy blocking two different pathways via inhibition of bromodomain and extraterminal domain (BET) and MEK pathways treating successfully NRAS-mutant and immune therapy-resistant melanoma [88].

#### PTPIP51 in Melanoma

PTPIP51 protein and its interactome in melanoma cells in relation to normal melanocytes derived from common nevi, as well as to melanocytes from dysplastic nevi revealed a characteristic profile within the three different entities. Dysplastic nevi represent an intermediate state between nevi and melanomas as they are morphologically and biologically intermediate between these two entities [89].

Despite massive changes in the molecular characteristics of melanocytes from normal nevi to dysplastic nevi and further on to malignant melanocytes, there is no significant change in the amount of PTPIP51 protein in these different stages. Yet, if the grade of Tyrosine 176 phosphorylation is analyzed, melanocytes from dysplastic nevi displayed the highest level in Tyr 176 phosphorylated PTPIP51 compared to the Tyr176 phosphorylation status of melanocytes derived from normal nevi, exhibiting the lowest phosphorylation level and when compared to the phosphorylation state in melanocytes from malignant melanomas displaying a tyrosine phosphorylation level laying in between. These findings are corroborated by the interaction profile of PTPIP51 and PTP1B, the phosphatase which is responsible for dephosphorylation of tyrosine 176, with the highest number of interactions in normal melanocytes and lowest number of interactions in melanocytes from dysplastic nevi and an interaction profile laying in between the aforementioned for malignant melanocytes. As described above, Tyr176 phosphorylation regulates the interaction of PTPIP51 with Raf. Solely when dephosphorylated at Tyr176 PTPIP51 can bind to Raf or 14-3-3β protein promoting MAPK signaling.

PTPIP51 affects the MAPK pathway by interacting with Raf-1 to stimulate downstream signaling. In melanocytes the interaction with the Raf-1/CRAF protein is relatively low. Nevertheless, there are quantitative differences for the three entities with lowest interaction numbers in malignant melanocytes and highest numbers in melanocytes from dysplastic nevi. Noteworthy, the interaction with BRAF is up to 100 times more numerous in the three different entities. PTPIP51/BRAF interaction is lowest in dysplastic nevi melanocytes, and somewhat higher in melanoma cells and highest in healthy melanocytes, where the number of interactions was five times higher compared to that of dysplastic nevi cells. This pattern corresponds well to that seen for PTPIP51 and PTP1B interaction and the measured phosphorylation status of PTPIP51. In addition, as indicated by the interaction profile of PTPIP51 and PKA, serine 212 phosphorylation which enhances the interaction of PTPIP51 and Raf, is much more reduced in dysplastic nevi and to a lesser degree in malignant melanoma cells as indicated by their lower interaction levels of PTPIP51/PKA in comparison to an almost doubled interaction seen in normal melanocytes. Noteworthy, serine212 phosphorylation promotes the activation of MAPK signaling by augmenting the interactome of PTPIP51, 14-3-3β and Raf-1.

These data argue for a counter-regulatory function of PTPIP51 in dedifferentiating melanocytes trying to reduce the stimulation by PTPIP51 interaction on BRAF level displaying lowest levels in dysplastic nevi and somewhat higher levels in malignant melanomas. The relative rise in the number of interactions in malignant melanocytes probably reflects the ongoing further dedifferentiation of the cells, thus leading to an increased MAPK signaling compared to dysplastic nevi and the highest stimulation to proliferate.

BRAF inhibitor resistance of melanoma can be a consequence of bypassing MAPK signaling via the PI3K-Akt pathway. Noteworthy, PTPIP51 is also involved in Akt signaling via a direct interaction with Akt protein. In untreated samples either from healthy control nevi or dysplastic nevi or from melanoma, melanocytes of the control display a high PTPIP51/Akt interaction, which is strongly reduced in samples from dysplastic nevi or melanoma. Akt stabilizes the communication site between mitochondria and endoplasmic reticulum. PTPIP51 likewise interferes in this mitochondria-ER relation via its VAPB interaction. The communication between both organelles is among other factors necessary for apoptosis [50]. Presumably, the reduced PTPIP51/Akt interaction is linked to the reduced apoptosic rate in dedifferentiated melanoma cells. An alternative mechanism for resistance is the activation of NF-κB [68]. NF-κB/PTPIP51 interaction is enhanced both in dysplastic nevi and melanoma cells with highest levels in the dysplastic nevi compared to the level in healthy melanocytes. The expression of PTPIP51 mRNA and protein is negatively regulated by RelA, thus affecting the apoptotic function of PTPIP51 [19].

To overcome therapy resistance in melanoma MEK inhibitors are tested in combination with a variety of drugs that use different approaches: inhibition of upstream Ras effectors, inhibition of PI3K-Akt-mTOR, inhibition of cell cycle regulators and activation of anti-tumor immunity but all seem to fail according to the existing cross-resistances [76].

**Figure 3:**
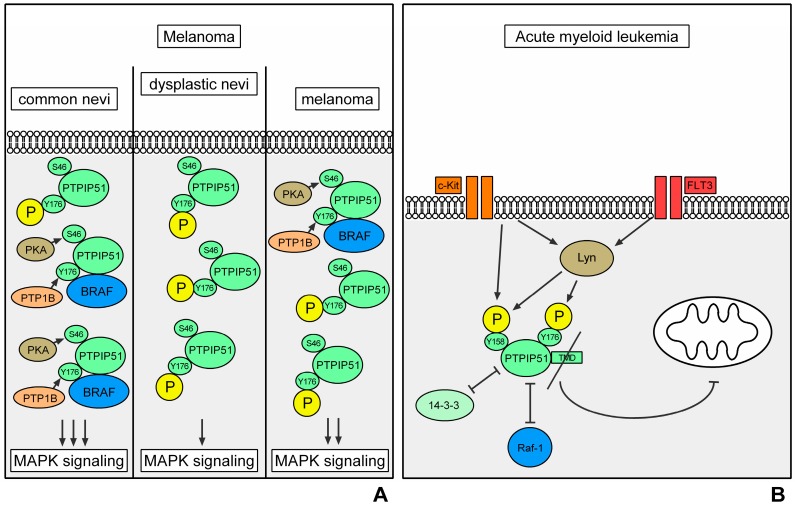
Regulation of PTPIP51 in melanoma. The left panel depicts the regulations of PTPIP51 in normal nevi. The phosphorylation level of the Tyr176 residue is low due to the high interaction with PTP1B. The phosphorylation of the Ser46 residue of PTPIP51 via PKA and the low phosphorylation level of the Tyr176 residue induce the formation of the PTPIP51/14-3-3β (not depicted)/BRAF complex and thereby a stimulation of the MAPK pathway. In the progression of the dysregulation of signaling as represented by the dysplastic nevi, the phosphorylation of PTPIP51 at Tyr176 is upregulated and thus inhibits the MAPK pathway stimulation of PTPIP51. This potentially mirrors a counter-regulation against the dysregulated growth and proliferation signaling in dysplastic nevi (middle panel). In melanoma cells the regulation of the phosphorylation and the interactions of PTPIP51 lie in between the normal nevi and the dysplastic nevi. This depicts the complete dysregulation of signaling since the counter-regulation of MAPK pathway via PTPIP51 phosphorylation is also deregulated (black arrows indicate a phosphorylation/interaction) (**A**); regulation of PTPIP51 interactions in acute myeloid leukemia. Activating mutations of the FMS like tyrosine kinase 3 (FLT3) receptor induce an activation of the Src family kinase Lyn. Lyn phosphorylates the Tyr158 and the Tyr176 residue of PTPIP51, which inhibit the formation of the Raf-1/14-3-3β/PTPIP51 complex and thus the MAPK pathway stimulation of PTPIP51. In acute myeloid leukemia blasts the N-terminus of PTPIP51 is missing. Therefore, the PTPIP51 protein does not contain the TMD. Due to the loss of the TMD a translocation to the mitochondrion is not possible and the apoptosis-inducing function of PTPIP51 is omitted (black arrows indicate a phosphorylation/activation, arrows with vertical bar as arrow head indicate an inhibition of interaction/activation) (**B**).

This strongly emphasizes the need for a better understanding of the MAPK/PTPIP51 interactome in melanoma (Figure 3A).

### 3.4. Acute Myeloid Leukemia

Leukemias are a group of heterogenous diseases with highly different malignant potential. In this context, two entities must be discriminated: acute leukemias and chronic leukemias. The later encompasses the chronic lymphocytic leukemia, which is classified as a non-Hodgkin lymphoma, and the chronic myelogenous leukemia. Both diseases are not curable but exhibit a prolonged course, e.g., with an eight years survival time of almost 87% for the chronic myeloid leukemia [90].

Acute leukemias are also subdivided into the two cell lineages, lymphocytic and myelogenous. Here, the group of acute myeloid leukemia (AML) is a highly malignant neoplasm exhibiting a near stable incidence over the last years with 3.7 affections per 100,000 persons and an age-dependent mortality of 2.7 to nearly 18 per 100,000 persons. The disease continuously shows two peaks in early childhood and later adulthood [91]. Moreover, despite advances in the therapeutic regimens of AML, the prognosis in the elderly who account for most new cases remains poor.

Interestingly, the prognosis as well as therapeutic decisions are tightly linked to specific cytogenetic and molecular alterations within the malignant transformed cells [92], encompassing microscopically detectable chromosome aberration, submicroscopic gene mutations and changes in gene expression. According to the underlying cytogenetic and mutational burden, patients can be classified into three prognostic categories: favorable, intermediate, and adverse [93]. Two types of cooperating mutations lead to alterations in self-renewal capacity, cellular differentiation, and cell survival of the AML blasts. So-called class II mutations affect transcription factors and lead to impaired differentiation. The class I mutations occur in RTKs, such as FLT3, c-KIT, or downstream effectors, such as Ras [94]. In consequence, they enhance cell survival and proliferation [95]. Interestingly, most of the alterations involve RTK, namely c-Kit and FLT3 [96].

The FMS-like tyrosine kinase 3 (FLT3) is a receptor tyrosine kinase which is expressed on the surface of CD34+ hematopoietic stem cells and other immature hematopoietic progenitors.

The receptor is classified as a type-1 transmembrane receptor tyrosine kinase encompassing several functional domains, e.g., an extracellular domain with Ig domains, TMD, and an intracellular tyrosine kinase domain with two kinase domains [97]. FLT3 is classified as a class III receptor tyrosine kinase besides the platelet-derived growth factor receptor (PDGFR), macrophage colony-stimulating factor receptor, and stem cell factor receptor (c-KIT). Upon activation with the cognate ligands the FLT3 tyrosine kinase couples to distinct downstream pathways, namely phosphatidylinositol-3 kinase (PI3K)-Akt pathway, the janus kinase (JAK)/signal transducer and activator of transcription (STAT) pathway and the MAPK pathway [97].

Approximately 30% of AML patients harbor some form of FLT3 mutation, which can be divided itself into two mutational classes: internal tandem duplications (FLT3/ITD mutations) in or near the juxtamembrane domain of the receptor and point mutations resulting in single amino acid substitutions involving the activation loop of the tyrosine kinase domain (FLT3/TKD mutations) [94]. Yet, these mutations are clinically and therapeutically challenging because of the nature of the mutation and the context in which it occurs [98]. Notably, FLT3/ITD mutation leads to uncontrolled cellular proliferation, survival, and differentiation through constitutive activation of FLT3 and the coupled downstream pathways, e.g., the consecutive activated MAPK pathway [97].

Besides the activation of whole signaling cascades, FLT3 is also able to phosphorylate specific signaling molecules, e.g., the Src family kinase Lyn. Compared to the wild type FLT3 receptor, FLT3/ITD displayed a higher affinity to bind to Lyn and the affinity was relative to the intensity of tyrosin phosphorylation of the receptor [99]. Lyn is known to play a critical role in leukemogenesis [95].

The other class III receptor tyrosine kinase, c-KIT, which also plays a pivotal role in AML, is expressed by myeloblasts in about 60% to 80% of patients: Therefore, next to the described FLT3 aberrations c-KIT harbors the most frequently observed activating RTK mutations in AML with an overall incidence of 17%. The mutations also encompass, for example, internal tandem duplications in c-Kit [100]. The coupled downstream pathways are identical to the FLT3 coupled pathways. Of note, Lyn activation can also be exerted by c-KIT [101].

#### PTPIP51 in AML

PTPIP51 is expressed in malignant transformed blasts of AML in an isotype specific manner. PTPIP51 protein isoforms could be traced with molecular weights of 13 kDa, 25 kDa, 38 and 52 kDa, respectively [102]. Using peptide sequence specific antibodies, only the peptide sequence for the C-terminal portion of PTPIP51 could be traced, whereas no staining of the N-terminal or the aa 114–129 protein sequence could be observed [102]. As reviewed by Brobeil et al. alternative splicing and the leaky scanning mechanism may build the base for the (disease) specific isoform expression of PTPIP51 [9]. The resulting protein sequence can lack distinct functional domains. In the case of AML, PTPIP51 lost the TMD located at the N-terminal portion of PTPIP51 [102], which is crucial for the mitochondrial binding and the apoptotic function of PTPIP51 [7]. In silico experiments using the Group-based Prediction System 2.1 (GPS 2.1) with the full-length protein sequence as input (SwissProt acc. no. Q96TC7) disclosed that PTPIP51 can be phosphorylated by Lyn, c-Src and c-Kit, respectively [9]. These interactions could be verified in AML blasts by using an in situ approach (DuoLink proximity ligation assay) [102]. Tyrosine 158 and 176 of PTPIP51 serve as phosphorylation residues for Lyn and c-Src, while c-Kit phosphorylation is limited to tyrosine 158 [9]. Interestingly, two functional domains of PTPIP51 are near these phosphorylation sites arbitrating the 14-3-3β binding and, therefore, the Raf-1 and MAPK signaling modulation [7]. The conserved region 1 spans aas 45 to 48 but is completely missing on the protein sequence of PTPIP51 in AML blast [7,102]. The conserved region 2, spanning aas 146–154, is near the tyrosine 158 and 176 residue [9]. As shown by HaCaT cell line experiments exposing the cells to EGF, the phosphorylation of these tyrosine residues leads to the disassembly of the PTPIP51/14-3-3β/Raf-1 complex resulting in a decreased stimulation of the MAPK pathway by PTPIP51 on Raf-1 level [11]. As FLT3 and c-KIT, both RTKs, are constitutive active in most AML cases, the physiological mechanism of regulating the MAPK stimulatory function of PTPIP51 may be still intact in AML blasts. This is also resembled by the high tyrosine 176 phosphorylation levels of PTPIP51 in AML blasts [102]. In none of the AML samples an interaction of PTPIP51 with Raf-1 could be traced due to the high tyrosine 176 phosphorylation levels, despite regions with small residues of normal hematopoiesis [102]. The probable apoptotic function of hyperphosphorylated PTPIP51 is omitted as the TMD is missing leading to uncontrolled proliferation by the constitutive activation of the MAPK by FLT3 and c-KIT [7,8,102]. Using the GPS 2.1 algorithm an enzyme-substrate relation between FLT3 and PTPIP51 could not be verified in contrast to the results gained for c-Kit [102]. PTPIP51 is co-located with Lyn and the interaction of both could be proved in situ by the DuoLink proximity ligation assay in AMLs blast [102]. Thus, c-Kit activity probably leads to the phosphorylation of tyrosine 158 residue and FLT3 mediated Lyn activation leads to phosphorylation of tyrosine 176 residue preventing PTPIP51 to bind 14-3-3β and in consequence modulating the MAPK activity in AML blasts. This inhibited interaction cannot be reversed by dephosphorylation of PTPIP51, as the main dephosphorylating enzyme PTP1B is absent in AML blasts [8,10,11,102].

As Dasatinib blocks Lyn activity with consecutive apoptosis in imatinib-resistant CML cells [103], the interaction blockage of PTPIP51 with the MAPK could probably be abolished with the initiation of apoptosis by administrating Dasatinib. Moreover, Dasatinib also binds to the c-KIT receptor suppressing its activity and promotes cellular apoptosis via activation of the caspase-dependent apoptotic pathway in AML blasts [104].

In summary, PTPIP51 displays a disease-related isoform expression in AML with loss of functional domains. Yet, the physiological regulation of the MAPK binding capacity of PTPIP51 seems to be intact. Thus, the involved signaling molecules of PTPIP51 can be directly targeted by small molecules to induce apoptosis in AML blasts (Figure 3B).

## 4. Summary

Protein-protein interactions are the foundation of all signaling events in normal cells as well as in dysregulated tumor cells. The framework for the correct procedure of signaling, the interconnection of different pathways and the appropriate subcellular localization is provided by scaffold proteins [105].

In this review, we highlight the central position of PTPIP51 within the dysregulated MAPK pathway signaling of several disparate diseases. The interactions and thus the functions of PTPIP51 are regulated by the serine and tyrosine phosphorylation status of PTPIP51 [8,9,10,11,12]. It exerts its main function in the MAPK pathway via the binding and stimulation of Raf proteins [7]. As seen in melanoma, the inhibition of the Raf stimulating effect of PTPIP51 can be used as a counter-regulatory mechanism against the BRAF deregulation within the sequence from normal nevi over dysplastic nevi to melanoma. Likewise, mechanisms were seen in breast cancer samples. Here, the observed high Tyr176 phosphorylation prevents the binding and thus the stimulation of Raf-1 via PTPIP51. In these tumors PTPIP51-related signaling represents a counter-regulation against the dysregulated growth and proliferation signaling (ongoing studies of our group). In insulin signaling, not the inhibition but the stimulation of the MAPK signaling via PTPIP51 represents the counter-regulatory mechanism against insulin resistance, showing the importance of the cellular setting in which the signaling takes place.

During the progression of tumorous diseases, the PTPIP51 regulating kinases and phosphatases often succumb alterations, in the form of activating mutations, overexpression or over-activation. C-Src represents a perfect example for tumor promoting and therapy resistance inducing kinases, which also regulates the phosphorylation of PTPIP51 [8,11,41,46,67]. The interaction of c-Src and PTPIP51 determines the sensitivity of Her2 amplified breast cancer cells towards EGFR-targeted TKIs [50]. The same interaction might be the reason for the anti-EGFR-therapy resistance of glioblastoma cells. A summary of the relevance of PTPIP51 as a diagnostic biomarker and as a therapeutic target in the various described diseases is presented in Table 1.

To sum up, PTPIP51 modulates the upmost position of the ERK pathway, the MAPKKK Raf-1. Furthermore, PTPIP51 crosslinks this signaling node with several tumor relevant RTKs, non-receptor tyrosine kinases and protein tyrosine phosphatases. PTPIP51 connects the MAPK pathway with several other signaling systems, e.g., the Akt and NF-κB signaling, which exceed the scope of this review. The precise understanding of the PTPIP51-related interactome in tumors is of the utmost interest and offers the possibility to understand dysregulated signaling systems and potential targetable signaling molecules. In particular, the interaction of PTPIP51 and c-Src seems to be of great relevance for therapy resistance mechanisms in several tumor entities and needs further investigation.

## Figures and Tables

**Table 1:** Summary table of the PTPIP51-related mechanisms of the various diseases and their implications on the role of PTPIP51 as a potential biomarker and targetable molecule.

Disease	PTPIP51-Related Mechanisms	Role of PTPIP51 as Diagnostic Biomarker	Targetable Molecule
Insulin Resistance	Transcriptional regulation of the IR via MAPK activation through the formation of the PTPIP51/14-3-3β/Raf-1 complex	expression of PTPIP51 negatively correlates with the grade of insulin sensitivity in mice	shifting PTPIP51 into MAPK signaling could enhance the transcription of IR and thus the insulin sensitivity
Breast Cancer	Sensitivity to EGFR/Her2 targeted TKIs depends on the formation of the Her2/c-Src/PTPIP51 complex	PTPIP51/PTP1B interaction positively correlates with the grading	Targeting the formation of the Her2/c-Src/PTPIP51 complex could overcome anti-EGFR/Her2 therapy resistances
Glioblastoma Multiforme	EGFR-targeted therapies are potentially bypassed via an enhanced interaction of c-Src and PTPIP51	PTPIP51 mRNA expression positively correlates with the grading of glioma	Targeting the PTPIP51/c-Src interaction could overcome anti-EGFR therapy resistances. Inhibition of the PTPIP51/14-3-3β interaction could unveil the TMD of PTPIP51 and promote the apoptosis-inducing functions of PTPIP51
Melanoma	Modulation of the serine and tyrosine phosphorylation of PTPIP51 via PKA and PTP1B induces a disease-stage-dependent alteration of the formation of the PTPIP51/14-3-3β/Raf-1 complex and thus the MAPK pathway activation	Phosphorylation and interaction profile of PTPIP51 is altered stage-dependently	Inhibition of PKA and PTP1B could reduce the interaction of PTPIP51 and BRAF and thus the MAPK stimulating effect of PTPIP51
Acute Myeloid Leukemia	Loss of the TMD of PTPIP51 inhibits the apoptosis-inducing functions of PTPIP51. Phosphorylation of PTPIP51 via Lyn and c-Kit prevents the PTPIP51-induced MAPK pathway activation	PTPIP51 is expressed in a disease-related isoform without TMD	
